# The achievement–dropout paradox: structural and performance predictors of youth-to-senior transition attrition in elite handball

**DOI:** 10.3389/fspor.2026.1868532

**Published:** 2026-06-10

**Authors:** Csilla Ildikó Filó

**Affiliations:** Department of Sports Science, Institute of Physiotherapy and Sports Science, Faculty of Health Sciences, University of Pécs, Pécs, Hungary

**Keywords:** athlete dropout, elite handball, homegrown players, structural reform, talent development, team performance, youth-to-senior transition

## Abstract

**Background:**

Dropout during the youth-to-senior transition represents a critical challenge in competitive team sports, yet it is often examined primarily at the individual level. While burnout frameworks provide an important theoretical lens for understanding how accumulated developmental stress may contribute to career discontinuation, dropout is a multi-determined outcome that also reflects structural, organisational, and opportunity-related constraints. Limited evidence exists on how structural reforms, organisational continuity, and competitive performance environments jointly shape transition outcomes in elite youth handball.

**Methods:**

This study analysed longitudinal registry data from 1,898 male handball players who completed U20 eligibility in Hungary between 2013 and 2023. Dropout was defined as termination of competitive handball following the U20 stage. Multivariable logistic regression and survival analyses were used to examine the association between dropout and birth cohort, exposure to a major competition reform, homegrown status, late-adolescent club mobility, and team competitive strength during the final U20 season.

**Results:**

Overall, 31.8% of players dropped out immediately after U20 completion, with dropout concentrated between ages 18 and 21. Exposure to the post-2017–18 competition structure was associated with significantly higher dropout risk (OR = 2.47, 95% CI 1.89–3.23). Homegrown players showed substantially lower dropout odds compared with non-homegrown players (OR = 0.18, 95% CI 0.13–0.25), while late-adolescent club mobility increased dropout risk (OR = 1.34, 95% CI 1.11–1.62). Team performance context exhibited a non-linear association with dropout: players from both low-performance (OR = 1.56, 95% CI 1.18–2.07) and high-performance U20 teams (OR = 1.41, 95% CI 1.05–1.89) showed elevated dropout risk relative to those from moderately performing teams.

**Conclusions:**

Dropout during the youth-to-senior transition in handball is a systemic phenomenon shaped by the interaction of structural exposure, organisational continuity, and competitive performance ecology. Because psychological burnout was not directly measured, findings are interpreted as associations rather than causal pathways. Balanced developmental environments and sustained homegrown pathways appear associated with stronger long-term retention. These findings highlight the need for talent development systems to align youth competition structures with sustainable senior integration capacity.

## 1 Introduction

The transition from youth to senior competition is widely recognised as one of the most vulnerable phases in athletic development, characterised by escalating performance demands, organisational uncertainty, and increasing psychosocial pressure. A substantial body of research has demonstrated that this period is associated with elevated levels of burnout symptoms and a heightened risk of dropout across youth sport systems (1). Burnout—understood as a syndrome of emotional exhaustion, depersonalisation, and reduced sense of accomplishment—has been proposed as one theoretical mechanism through which accumulating developmental stress may contribute to career discontinuation, particularly during critical transitions. However, dropout is a multi-determined behavioural outcome that may also reflect structural constraints, educational choices, deselection, and opportunity limitations, and should not be equated with burnout in the absence of direct psychological measurement. In team sports such as handball, where structured talent pathways, early selection, and competitive exposure are particularly pronounced, the youth-to-senior transition represents a decisive bottleneck for long-term athlete retention.

Established theoretical frameworks offer complementary perspectives on the mechanisms underlying dropout during this transition. Self-Determination Theory (2) highlights how thwarted needs for autonomy, competence, and relatedness may undermine intrinsic motivation and increase vulnerability to disengagement. Achievement Goal Theory (3) draws attention to the role of performance climates and ego-threatening environments in generating evaluative pressure. Athlete burnout frameworks (4) further emphasise how chronic stress exposure—accumulating across developmental stages—may erode athletes’ psychological resources, ultimately contributing to withdrawal. Importantly, these frameworks converge on the insight that dropout risk is not solely determined by individual characteristics but is shaped by the developmental environment itself: the structure of competition systems, the quality of organisational support, and the balance between challenge and opportunity. Prior empirical work has demonstrated that sport-specialising pathways are consistently associated with higher burnout risk compared with sport-sampling trajectories (1), reinforcing the relevance of systemic conditions as antecedents of attrition.

More recent evidence suggests that the psychosocial conditions surrounding the youth-to-senior transition have become increasingly complex. Contemporary research outside sport contexts highlights the growing influence of perceived parental expectations on adolescents’ developmental decisions, with choices at critical junctures often shaped more strongly by perceived external demands than by objective competence indicators (5). Within organised youth sport, parental involvement has been shown to function simultaneously as a protective and a risk factor, with heightened evaluative behaviours associated with elevated stress and potentially contributing to withdrawal from sport (6). These patterns are consistent with broader structural shifts in which athletic performance and educational attainment are evaluated in parallel, generating competing demands on young athletes. While these psychosocial dynamics provide important contextual background, the present study does not directly examine psychological variables; rather, they inform the interpretation of structural and organisational factors that may create or amplify such pressures at the system level.

Handball-specific research provides further contextual evidence. Barić (7) identifies systematic discrepancies between athletes’ and coaches’ perceptions of motivational climate, suggesting potential misalignment in team environments that may be associated with reduced well-being. Kovács et al. (8) report that youth handball players’ perceptions of parental involvement vary across participation stages and are associated with psychosocial strain, indicating that family-related pressures interact dynamically with sport-specific stressors during the developmental pathway. Research on burnout in handball has further demonstrated that psychological overload may emerge early: Santos-Afonso et al. (9) document clinically relevant burnout symptoms among elite youth handball players, while González-Hernández et al. (10) show that strong commitment coupled with fear of failure is associated with higher burnout levels, illustrating how dedication can become a risk factor in evaluative environments. These findings support the theoretical premise that high-performance developmental contexts may carry unintended psychological costs—a mechanism that ecological models of talent development (11) associate with imbalanced challenge-support ratios. Crucially, however, none of these studies employed register-based designs capable of linking such psychological conditions to verified career discontinuation at population level.

Despite these theoretical and empirical contributions, a critical gap remains in the existing literature. Most studies on burnout and dropout rely on cross-sectional designs, self-reported psychological measures, and short-term indicators of disengagement. Even prospective cohort studies, such as that by Nielsen et al. (12), primarily focus on motivational predictors of continued participation rather than objectively verified career termination. Consequently, the evidence base linking burnout-related theoretical frameworks to actual, population-level dropout during the youth-to-senior transition remains limited, particularly in team sport contexts. What is largely absent are register-based longitudinal analyses capable of examining how structural, organisational, and competitive performance conditions—which may amplify or buffer the psychosocial pressures described above—are associated with objectively observed career continuation or discontinuation at the system level.

Cohorts entering the youth-to-senior transition after 2018 have experienced a markedly altered developmental context, shaped by structural and institutional changes rather than by inherent generational characteristics. The expansion of competition systems, intensified parental involvement, educational-sport role conflict, and the disruptions associated with the post-2020 period represent contextual conditions that differentiate recent cohorts from those examined in earlier research. These shifts suggest that burnout-dropout models derived from pre-2020 cohorts may provide an incomplete account of contemporary attrition patterns (5, 6). Critically, however, interpreting these contextual shifts requires structural and organisational data rather than self-reported psychological measures—which is precisely the approach adopted in the present study.

The extant literature thus underscores the necessity for longitudinal, register-based studies that capture objectively verified career discontinuation during the youth-to-senior transition. Such studies are particularly warranted in handball, where highly structured developmental pathways and recent organisational shifts provide a unique opportunity to examine how structural reforms, organisational continuity, and competitive performance context are associated with actual attrition outcomes—independently of self-reported psychological states.

A recent longitudinal analysis using the same national register database (13) examined structural predictors of youth-to-senior transition outcomes in Hungarian male handball, identifying generational load, organisational maturity, and pathway accessibility as key determinants of career development. While that study provided a systemic overview of structural effects, it did not examine dropout in relation to burnout-related theoretical frameworks, nor did it assess post-2018 cohort conditions, competitive performance context, or specific career trajectory patterns. The present study extends this work by analysing these dimensions explicitly, using objectively observed career outcomes as the dependent variable while drawing on burnout and ecological talent development frameworks as interpretive lenses rather than measured constructs.

The investigation of retention and attrition in Hungarian male handball carries particular relevance, as the sport's domestic institutional framework underwent a significant structural shift in 2016. In that year, the Hungarian Handball Federation restructured the national youth championship system: the previous single-group national elite league (comprising 12–14 teams) was replaced by a two-group regional system consisting of 32 teams. This degree of competitive expansion is rare by international standards and resulted in a radical increase in the capacity of the talent development pipeline. Consequently, significantly more players reached the national youth elite level; however, the absorption capacity of adult leagues (NB I, NB I/B, NB II) did not keep pace with this expansion.

This structural tension—namely, increased youth output vs. stable, limited adult absorption capacity—creates a classic “bottleneck effect” in the sense of international literature. While the apex of the sporting pyramid is inherently narrow in every country, the structural expansion in Hungarian male handball was temporally concentrated and systemic. This potentially highlights sharp cohort differences between players entering the youth ranks before and after 2016. It is expected that transition probabilities for post-2016 cohorts will deteriorate, as the “exit group” they comprise is larger and more heterogeneous, while the number of adult teams and their rotational requirements have remained stagnant.

International longitudinal research on talent development also highlights that transition success is heavily influenced by whether an athlete is “homegrown” within a specific club's system. The stability of intra-club developmental pathways, technical-tactical consistency, and the continuity of coaching and professional relationships typically reduce the likelihood of dropout and provide greater protection during critical age-related transitions. In contrast, non-homegrown players more frequently find themselves on the periphery, receive less club-specific support, and are more vulnerable when moving into the adult category.

Utilizing a unique database—comprising the complete career data of 2,265 Hungarian male youth players—the present study examines the structural effects of the 2016 competition system shift, dropout trends, survival curves, cohort differences, and the role of homegrown status. A major strength of this investigation is that it covers the entire population of players born between 1993 and 2007, enabling systemic conclusions through a non-sampling-based approach.

Similar transition bottlenecks have been documented in other European handball systems, including Denmark, Germany, and France, where broad youth participation coexists with highly selective senior-level integration, despite differing organisational and market structures.

### 1.1 Study aims and hypotheses

#### 1.1.1 Study aims

Building on emerging evidence that the youth-to-senior transition in sport is shaped not only by individual psychological processes but also by broader structural, organisational, and cohort-specific conditions, the present study seeks to provide a system-level, register-based analysis of career retention and attrition in Hungarian male handball. By leveraging a full-population longitudinal dataset covering 2,265 players born between 1993 and 2007, this study aims to deepen current understanding of how structural reforms, generational conditions, and club-level developmental environments influence the likelihood of sustained participation vs. dropout during the transition to senior competition.

The specific aims are:

1. To examine dropout patterns and survival trajectories during the youth-to-senior transition using objectively observed career outcomes.
2. To assess the impact of the 2016 Hungarian competition reform, which doubled the size of the national youth elite league, on dropout risk and cohort-level instability.
3. To analyse the role of developmental instability, operationalised through club mobility patterns in late adolescence, in predicting dropout.
4. To evaluate the protective role of homegrown status, as a marker of intra-club developmental continuity and organisational support.
5. To investigate post-2018 and post-2020 cohort effects, considering the intensification of parental expectations, sport-school conflicts, and COVID-19–related disruptions documented in recent literature.

#### 1.1.2 Hypotheses

H1 — Transition Timing Hypothesis: Dropout risk will be highest during the initial phase of the youth-to-senior transition, with the steepest decline in survival between ages 18 and 21. A career termination events the majority will occur during the first three seasons following youth-level eligibility.

H2 — Developmental Instability Hypothesis: Players exhibiting higher developmental instability—operationalised through multiple club changes during late adolescence—will show significantly higher dropout risk than players following a stable, continuous developmental path.

H3 — Organisational Pathway Hypothesis: Belonging to organisations that provide a structured, direct senior pathway—and being classified as a “homegrown” player—will be associated with significantly lower dropout probability. Intra-club developmental continuity, stable coaching relationships, and internal advancement routes are expected to act as protective factors.

H4 — Structural Reform Cohort Hypothesis: Players entering the youth system after the 2016 competition reform (BirthYear ≥ 1999) will exhibit significantly higher dropout rates than pre-2016 cohorts, even after controlling for individual and organisational variables.

H5 — Structural Overexpansion and Absorption Bottleneck Hypothesis:The expansion of the national U20 competition system implemented by the Hungarian Handball Federation in the 2016–2017 season, which increased the number of participating teams from 16 to 32, substantially enlarged the pool of players reaching the end of youth eligibility without a parallel expansion of senior-level absorption capacity. It is therefore hypothesised that dropout rates increased markedly from the 2016–2017 season onward, as successive cohorts aged out of the youth system while facing limited opportunities for placement in adult teams. This effect is expected to manifest as a progressive rise in post-U20 dropout across seasons, reflecting a structural bottleneck at the youth-to-senior transition rather than a short-term or cohort-specific disruption.

H6 — Generational Instability Hypothesis: Post-2018 and post-2020 cohorts will demonstrate shorter survival times and higher transition hazard values than earlier-cohort athletes, due to intensified parental expectations, heightened evaluative pressure, and increased organisational uncertainty documented in contemporary studies.

H7 — Trajectory Differentiation Hypothesis: It is expected that dropout will not occur uniformly across athletes but will follow systematically divergent developmental trajectories, with transition outcomes shaped by the timing and pattern of competitive participation, the degree of developmental stability, and structurally induced constraints on progression.

H8 — Achievement–Dropout Paradox Hypothesis: Dropout will exhibit a non-linear association with the competitive success context: athletes from both highly successful and poorly performing U20 teams will display elevated dropout risk compared to players from moderately successful developmental environments.

## 2 Methods

### 2.1 Study design

This study adopted a retrospective, register-based longitudinal design to examine the structural, organisational, and contextual determinants of youth-to-senior transition outcomes in Hungarian male handball. The analysis was based on full-population administrative data covering all athletes who participated in the first-class U20 national youth championship system between 2013 and 2023. Before the organisational reform implemented for the 2017–18 season, this age category was officially referred to as the “Junior” or “Youth” championship. The reform introduced a two-group (East–West) structure with a substantially expanded number of participating teams. By relying on population-level administrative data rather than sampled observations, the study provides system-level insights into developmental patterns and dropout risk in Hungarian handball.

### 2.2 Data sources

Individual-level longitudinal records were obtained from the Hungarian Handball Federation's registration database, which includes season-by-season information on player affiliations, youth and senior competition participation, and highest competitive level attained. The analytic dataset comprised 2,265 players born between 1993 and 2007, representing the entire population of athletes who reached U20 eligibility during the study window. Each athlete was tracked from initial youth registration until either verified discontinuation of competitive participation or the end of the observation period in 2023. Right-censoring was applied for athletes who remained active beyond this point.

To characterise the competitive environments in which youth players developed, team performance data were extracted from official league tables for all U20 national championships between 2013 and 2023. These year-specific tables included final rankings, points earned, goal differences, goals scored and conceded, and match outcomes for every team in the national youth competition system, including the East–West split introduced in 2017–18.

### 2.3 Variables

#### 2.3.1 Dropout classification

Dropout was defined as an objectively verifiable termination of competitive handball following the completion of U20 eligibility. A seven-level hierarchical competitive classification captured post-youth trajectories. Level 1 represented continued participation in the top-tier senior league (NB I), Level 2 participation in NB I/B, and Level 3 in NB II. Level 4 denoted county-level adult competition, while Level 5 captured senior continuation through foreign professional contracts. These levels collectively constituted successful youth-to-senior transitions. Level 6 indicated withdrawal immediately after the U20 stage and thus operationalised dropout as the primary binary outcome. Level 7 reflected athletes who had not yet completed U20 eligibility by the end of data collection in 2023 (i.e., players born in 2005–2007 who remained active in the youth system). These cases (*n* = 367) were excluded from all inferential analyses because their transition outcome had not yet been observed, which would constitute right-truncation bias rather than the right-censoring applied to players whose senior career was ongoing at follow-up. Including them would artificially inflate the denominator and underestimate dropout rates for recent cohorts. The final analytic sample comprised 1,898 players with fully resolved transition outcomes.

#### 2.3.2 Cohort indicators

A structural cohort variable distinguished players who experienced youth competition under the reorganized system introduced in 2017–18. Because athletes born in 2000 or later entered the U20 category fully within the post-reform structure, this group was identified as the post-reform cohort; those born before 2000 formed the pre-reform reference cohort. Additional temporal cohort indicators captured broader shifts in developmental context, including athletes entering adolescence after 2018 (associated with intensifying academic–sport role conflict and parental expectations) and players transitioning after 2020 (exposed to COVID-19–related disruptions).

#### 2.3.3 Developmental instability and homegrown Status

Developmental instability was captured through late-adolescent club mobility, operationalised as the number of club changes recorded between the U17 and U20 registration seasons. Each unique club affiliation recorded in the federation database within this window was counted; mid-season transfers resulting in two registrations within a single season were identified through chronological harmonisation and counted as a single change. Homegrown status was defined as a binary indicator reflecting whether an athlete made his senior competitive debut within the same club organisation in which his U20 development primarily took place. Players who spent the majority of their U17–U20 seasons at one club but subsequently transferred and debuted senior competition at a different club were classified as non-homegrown. Athletes with documented temporary loan arrangements who returned to and debuted with their parent club were classified as homegrown, as these cases reflect intra-organisational continuity despite temporary absence. Dual development pathways (simultaneous registration at youth and senior level for different clubs) were resolved by assigning primary affiliation to the club recording the greater number of competitive appearances in the relevant season. This operationalisation reflects intra-organisational continuity and the availability of internal senior pathway support structures.

#### 2.3.4 Team competitive strength

To contextualise individual developmental environments, a Team Competitive Strength Index (TCSI) was derived from season-specific U20 league tables. Ranking position (reverse-scored), points earned, and goal difference were standardised within season and combined into a continuous index reflecting each team's competitive strength. This index was assigned to each athlete based on the team represented in their U20 season(s), providing a consistent measure of developmental performance context.

### 2.4 Data processing

Individual-level and team-performance datasets were harmonised through season-specific team identifiers. Because the youth league underwent structural changes in 2017–18, all performance variables were standardised within season to ensure comparability across formats (single-group pre-reform vs. East–West post-reform). Time-to-event values were computed as the difference between the final competitive season and the birth year, yielding an age-anchored measure of retention duration. Data cleaning included verification of date irregularities, harmonisation of club names across rebranding or sponsorship changes, and removal of players with incomplete transition opportunities. Regarding missing data: the registry database was administratively complete for all variables derived directly from registration records (club affiliation, competition level, birth year). Missing values were present only for the Team Competitive Strength Index in cases where a team did not complete a full season due to withdrawal or administrative exclusion (*n* = 23 player-seasons, 1.2% of the analytic sample). These cases were excluded listwise from models including TCSI; sensitivity analyses confirmed that their exclusion did not materially alter the parameter estimates for other predictors. Potential confounding variables not captured in the administrative dataset—including injury history, playing position, socioeconomic background, and educational demands—could not be controlled for and represent a limitation of the registry-based design.

### 2.5 Quality control

Multiple quality assurance steps ensured accuracy and coherence. Registration histories were cross-checked with external competition reports to validate career endpoints. Team performance tables were manually verified across seasons for consistency in match counts, points allocation systems, and ranking rules. Duplicate records resulting from mid-season transfers were resolved through chronological harmonisation. All derived variables (mobility, cohort assignment, TCSI) were validated through internal consistency checks and season-by-season manual audits.

### 2.6 Statistical analysis

Analyses proceeded in three stages. First, descriptive statistics summarised transition outcomes, cohort distributions, team-performance environments, and developmental instability profiles. Logistic regression models then estimated the associations between dropout and structural (reform cohort, post-2018 and post-2020 periods), organisational (homegrown status), contextual (TCSI), and individual (birth year, club mobility) predictors. Prior to model estimation, key regression assumptions were evaluated. Independence of observations was satisfied by the population-level, non-clustered sampling frame. The absence of perfect separation was confirmed by inspection of cross-tabulations. Multicollinearity was assessed using variance inflation factors (VIF); all predictors returned VIF values below 3.0, indicating no problematic collinearity. Model fit was evaluated using the Hosmer–Lemeshow goodness-of-fit test and Nagelkerke pseudo-R^2^. Odds ratios are reported with 95% confidence intervals; statistical significance was set at *α* = .05. Effect sizes are interpreted in the context of the full OR with confidence intervals rather than *p*-values alone, given the population-level sample size where modest effects may achieve statistical significance. Age-anchored retention patterns were examined using Kaplan–Meier survival functions stratified by cohort and team performance context. The proportional hazards assumption was not formally invoked, as Kaplan–Meier estimation is non-parametric. All analyses were conducted in Python (version 3.11) using the statsmodels, lifelines, and pandas libraries.

### 2.7 Ethical considerations

The study relied exclusively on administrative registry data available through the Hungarian Handball Federation. All data were anonymised before analysis, and no personally identifying information was used. The study complied with national data-protection regulations and the ethical standards required for research involving anonymised secondary datasets.

## 3 Results

### 3.1 Sample characteristics

The final analytic sample consisted of 1,898 male handball players who completed their U20 eligibility during the observation period. Of these, 603 players terminated competitive handball immediately after the U20 stage, corresponding to an overall dropout rate of 31.8%, while 1,295 players (68.2%) continued into senior competition (Table 1).

**Table 1:** Sample characteristics and youth-to-senior transition outcomes.

Characteristic	*N*	%
Total sample	1,898	100.0
Transition outcome		
Senior continuation (Levels 1–5)	1,295	68.2
Dropout after U20 (Level 6)	603	31.8
Cohort		
Pre-reform cohort (BirthYear < 2000)	774	40.0
Post-reform cohort (BirthYear ≥ 2000)	1,124	60.0
Homegrown status		
Homegrown players	485	25.0
Non-homegrown players	1,413	75.0
Team performance context (last U20 season)		
Low TCSI	633	33.3
Medium TCSI	632	33.3
High TCSI	633	33.4

Percentages are calculated relative to the total analytic sample (*N* = 1,898). Dropout was defined as termination of competitive handball following completion of U20 eligibility (Level 6).

### 3.2 Prevalence and cohort distribution of dropout

Dropout prevalence varied substantially across birth cohorts. Among players born before 1997, dropout remained below 20%, whereas a pronounced increase was observed from the 1997 cohort onward. In the post-reform cohort (BirthYear ≥ 2000), dropout reached 41.6%, compared with 24.9% in pre-reform cohorts.

Team performance categories represent terciles of the Team Competitive Strength Index (TCSI) based on the final U20 season.

### 3.3 Predictors of dropout: multivariable logistic regression

Results of the multivariable logistic regression model are presented in Table 2. Later birth year was associated with a significantly increased likelihood of dropout (OR = 1.21, 95% CI 1.15–1.27, *p* < 0.001).

**Table 2:** Multivariable logistic regression predicting dropout after U20.

Predictor	OR	95% CI	*p*
Birth year	1.21	1.15–1.27	<0.001
Homegrown status (yes vs. no)	0.18	0.13–0.25	<0.001
Post-reform cohort (≥2000)	2.47	1.89–3.23	<0.001
Developmental instability (club mobility)	1.34	1.11–1.62	0.002
Team performance context (ref.: Medium TCSI)			
Low TCSI	1.56	1.18–2.07	0.002
High TCSI	1.41	1.05–1.89	0.021

Odds ratios (OR) were estimated using multivariable logistic regression. Dropout was defined as termination of competitive handball following completion of U20 eligibility (Level 6). Medium team competitive strength (TCSI) served as the reference category. OR > 1 indicates increased dropout risk, whereas OR < 1 indicates reduced dropout risk.

Homegrown status emerged as a strong protective factor, as homegrown players showed substantially lower dropout odds compared with non-homegrown players (OR = 0.18, 95% CI 0.13–0.25, *p* < 0.001).

Exposure to the post-reform U20 competition structure was independently associated with higher dropout risk (OR = 2.47, 95% CI 1.89–3.23, *p* < 0.001). Developmental instability, operationalised as late-adolescent club mobility, was also positively associated with dropout (OR = 1.34, 95% CI 1.11–1.62, *p* = 0.002).

Inclusion of team-level performance context further improved model fit. Compared with players from moderately performing teams, athletes from both low-performance (OR = 1.56, 95% CI 1.18–2.07, *p* = 0.002) and high-performance environments (OR = 1.41, 95% CI 1.05–1.89, *p* = 0.021) exhibited elevated dropout risk.

The multivariable model demonstrated acceptable fit (Hosmer–Lemeshow *χ*^2^ = 8.43, df = 8, *p* = .39) and explained a meaningful proportion of variance in dropout (Nagelkerke pseudo-R^2^ = 0.31). The incremental inclusion of TCSI improved model fit (*Δ*Nagelkerke pseudo-R^2^ = 0.018).

### 3.4 Timing of attrition: survival analysis

Kaplan–Meier survival analysis indicated that dropout was concentrated in early adulthood. Survival probability was 0.93 at age 18, declined to 0.81 at age 19, and further decreased to 0.77 at age 20. By ages 21–22, survival stabilised at approximately 0.73, indicating that nearly one-quarter of players exited competitive handball within three seasons following U20 completion.

### 3.5 Seasonal trends in dropout rates

Season-specific dropout rates revealed a pronounced temporal pattern across the observation period (2013/14–2022/23). In the early seasons of the study window, dropout rates remained relatively low and stable, ranging from 6% in 2013/14 to 9% in both 2014/15 and 2015/16.

A marked increase was observed from the 2016/17 season onward, when dropout rose abruptly to 19%, coinciding with the first cohorts affected by the expansion of the national U20 competition system. In the subsequent seasons, dropout rates continued to increase progressively, reaching 20% in 2017/18, 23% in 2018/19, and 28% in both 2019/20 and 2020/21. In the final seasons of the observation period, dropout stabilised at a high level, with rates of 29% in 2021/22% and 30% in 2022/23.

**Figure 1:**
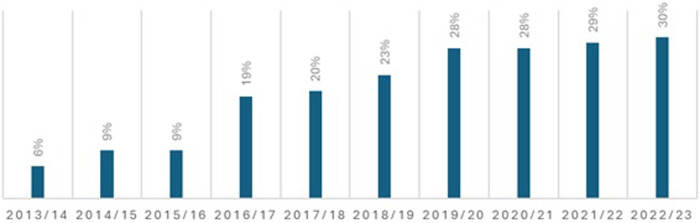
Seasonal dropout rates following U20 completion (2013–2023). The figure illustrates a marked increase in dropout rates coinciding with the expansion of the U20 competition system, followed by a progressive rise indicating structural saturation at the youth to senior transition.

Overall, the seasonal distribution indicates a clear shift from low and stable dropout rates prior to the structural reform toward substantially higher and progressively increasing dropout levels in the post-reform period. Rather than a single transient disruption, the observed pattern reflects a sustained elevation in dropout across successive cohorts reaching the end of U20 eligibility. These seasonal trends are illustrated in Figure 1.

### 3.6 Survival patterns across team performance contexts

Survival curves stratified by team competitive strength revealed distinct retention trajectories across the transition period. Players from moderately performing teams consistently exhibited more stable retention compared with those from both high-performance and low-performance environments, particularly during the critical 18–21 age window.

## 4 Discussion

### 4.1 Findings in context

The present study provides a system-level analysis of dropout during the youth-to-senior transition in Hungarian male handball, integrating structural, organisational, and performance-context factors. Four principal findings emerge. First, dropout is highly concentrated in early adulthood, particularly between ages 18 and 21, confirming the transitional nature of attrition. Second, younger cohorts exposed to the post-2017–18 competition reform exhibit substantially higher dropout risk. Third, homegrown developmental continuity emerges as the strongest protective factor identified. Fourth, dropout probability follows a non-linear pattern across team performance contexts, with elevated risk in both low- and high-performance U20 environments. Together, these findings indicate that dropout cannot be understood solely as an individual failure or lack of talent, but reflects the interaction between structural conditions, organisational pathways, and competitive environments. For interpretive purposes, it is important to distinguish between burnout as a psychological syndrome (emotional exhaustion, depersonalisation, reduced sense of accomplishment), temporary withdrawal, deselection, career transition, and permanent sport dropout: the present study observes only the last of these as a registry-based outcome, and cannot determine which underlying processes are operative in individual cases. Burnout frameworks and ecological models of athlete development provide useful interpretive lenses for the observed patterns; however, because psychological states were not directly measured, all interpretations are associative rather than causal, and dropout is treated throughout as a multi-determined behavioural outcome.

### 4.2 Structural reform and transition pressure

The increased dropout risk observed among post-reform cohorts suggests that the 2017–18 expansion of the U20 competition system altered the balance between participation opportunities and sustainable transition capacity. While structural expansion may enhance access and visibility at youth level, it can simultaneously intensify selection pressure at the point of senior transition, particularly when senior roster capacity does not expand proportionally. Similar dynamics have been reported in other European talent systems, where broad youth participation coexists with increasingly narrow senior opportunities, leading to heightened transitional attrition (14, 15). From a developmental systems perspective, such reforms may shift the timing of selection rather than eliminate it, resulting in delayed but more abrupt dropout during early adulthood. The season-specific dropout trends are consistent with a structural overexpansion mechanism: the enlarged youth competition system produced a growing number of players reaching the end of U20 eligibility without a corresponding increase in senior-level absorption capacity, and the persistence of elevated dropout rates across successive seasons suggests cumulative system-level pressure rather than a single exogenous disruption. These findings highlight how centrally coordinated competition reforms can unintentionally reshape transition dynamics when youth-level expansion is not matched by senior-level capacity.

### 4.3 Organisational continuity and the protective role of homegrown development

One of the strongest associations identified in this study is the protective role of homegrown status. Players developed within a club's internal pathway showed markedly lower dropout odds compared with non-homegrown players, even after controlling for cohort, age, mobility, and team performance context. This finding aligns with research highlighting the importance of organisational embeddedness, relational stability, and institutional trust in sustaining athlete careers (16, 17). Homegrown players are more likely to benefit from informal support networks, clearer role expectations, and greater tolerance for transitional underperformance—conditions that may buffer the stress associated with senior entry, consistent with Self-Determination Theory's emphasis on relatedness and competence support (2). Importantly, this effect persists independently of team performance, suggesting that continuity and organisational belonging may be as critical as competitive success in sustaining career progression.

### 4.4 Competitive performance context, developmental instability, and the achievement–dropout paradox

A central contribution of this study is the identification of a non-linear association between team competitive strength and dropout risk. Players developing in moderately performing U20 teams exhibited the lowest dropout probability, whereas both low-performance and high-performance environments were associated with elevated attrition. In low-performance environments, limited success may reduce motivation, perceived future opportunities, and institutional investment, increasing the likelihood of disengagement (4). Conversely, in high-performance environments, intensified competition, early role specialisation, and constrained senior access may be associated with exclusion or disengagement despite high objective performance (18, 19). Moderately performing teams may represent a developmental “optimal challenge” zone where demands are sufficiently high to foster progression but not so extreme as to undermine long-term sustainability—an interpretation consistent with ecological models of athlete development emphasising balance between challenge and support (11). This curvilinear pattern is what we term the achievement–dropout paradox: neither success nor failure at youth level reliably predicts retention, and both extremes of the performance spectrum are associated with comparable transition risk.

The positive association between late-adolescent club mobility and dropout further reinforces the importance of environmental stability during the transition phase. Frequent club changes may reflect unsuccessful attempts to secure senior opportunities, misalignment between expectations and reality, or reactive decision-making under selection pressure. Prior studies have shown that excessive mobility during late adolescence is associated with weaker identity formation, reduced social support, and increased dropout risk (20, 21). In this context, mobility appears less as a strategic career move and more as a behavioural marker of developmental instability, potentially reflecting the same structural pressures—reform-induced bottlenecks, limited senior pathways—that drive elevated dropout in the post-reform cohort more broadly.

### 4.5 Implications, limitations, and future directions

The findings carry important implications for federations, clubs, and practitioners. Structural reforms aimed at expanding youth competition should be accompanied by parallel investments in senior transition capacity, including reserve teams, loan systems, and flexible entry pathways into adult competition. Federations should consider monitoring dropout as a system-level indicator rather than an individual outcome, integrating longitudinal tracking into talent development evaluation frameworks. At the club level, the strong protective effect of homegrown status underscores the developmental value of long-term internal pathways and relational stability. Homegrown development should be viewed not only as a sporting or financial strategy but as a retention-enhancing mechanism. The non-linear association between team performance and dropout further suggests that youth success metrics alone are insufficient indicators of developmental quality: clubs operating at both extremes of the performance spectrum should be particularly attentive to transition support. For coaches and practitioners, the results emphasise the importance of managing expectations and developmental timing during late adolescence, where mobility is more indicative of instability than optimisation.

Several limitations should be acknowledged. The study relies on administrative registry data, which capture objective participation outcomes but not subjective motivations, psychological states, injury history, or educational trajectories. Because burnout was not directly measured, dropout should be interpreted as a behavioural outcome potentially influenced by a range of factors—including educational choices, injury, deselection, and opportunity structures—rather than as a direct indicator of burnout. Female pathways were not included, limiting generalisability across genders. Team performance was operationalised at the season level and may not capture within-team role differentiation or individual exposure. The study design is observational, and the associations reported do not permit causal inference; in particular, the reform effect cannot be fully isolated from concurrent contextual influences such as COVID-19 disruptions, educational pressures, and broader societal changes affecting youth sport participation. Future research could integrate qualitative methods to examine individual decision-making processes, extend the model to female and mixed-gender systems, incorporate direct measures of burnout and psychological well-being, and explore how educational and labour-market transitions interact with sporting dropout at the system level.

## 5 Conclusion

This study demonstrates that dropout during the youth-to-senior transition in Hungarian male handball is not a marginal or purely individual phenomenon, but a structurally embedded outcome shaped by interacting developmental conditions. Dropout is highly concentrated in early adulthood, disproportionately affects younger and post-reform cohorts, and is strongly associated with organisational continuity and competitive performance context. Homegrown development emerges as the most robust protective factor, while late-adolescent mobility and exposure to both low- and high-performance U20 environments are associated with elevated transition vulnerability.

The results challenge linear assumptions about youth success and senior sustainability: competitive excellence at U20 level does not guarantee long-term retention, nor does limited performance inevitably lead to dropout. Instead, the findings point to the importance of balanced developmental environments that align performance demands with realistic transition opportunities. Because the study design is observational and psychological states were not directly measured, these associations should not be interpreted as causal pathways; rather, they highlight structural and organisational conditions that appear systematically associated with attrition at the system level.

Taken together, the evidence supports a systemic interpretation of dropout: sustainable talent development depends not only on identifying high performers, but on designing pathways that integrate structural capacity, organisational stability, and performance ecology across the critical youth-to-senior transition.

## Data Availability

The raw data supporting the conclusions of this article will be made available by the authors, without undue reservation.
